# A Compact Polarization MMI Combiner Using Silicon Slot-Waveguide Structures

**DOI:** 10.3390/mi14061203

**Published:** 2023-06-06

**Authors:** Omer Brand, Benjamin Wolftson, Dror Malka

**Affiliations:** Faculty of Engineering Holon, Institute of Technology (HIT), Holon 5810201, Israel; sabosalob@gmail.com (O.B.);

**Keywords:** FV-BPM, MMI, slot waveguide, polarization, combiner

## Abstract

The study of designing a compact transverse electric (TE)/transverse magnetic (TM) polarization multimode interference (MMI) combiner based on silicon slot-waveguide technology is proposed for solving the high demands for high-speed ability alongside more energy power and minimizing the environmental impact of power consumption, achieving a balance between high-speed performance and energy efficiency has become an important consideration in an optical communication system. The MMI coupler has a significant difference in light coupling (beat-length) for TM and TE at 1550 nm wavelength. By controlling the light propagation mechanism inside the MMI coupler, a lower order of mode can be obtained which can lead to a shorter device. The polarization combiner was solved using the full-vectorial beam propagation method (FV-BPM), and the main geometrical parameters were analyzed using Matlab codes. Results show that after a short light propagation of 16.15 μm, the device can function as TM or TE combiner polarization with an excellent extinction ratio of 10.94 dB for TE mode and 13.08 dB for TM mode with low insertion losses of 0.76 dB (TE) and 0.56 dB (TM) and the combiner function well over the C-band spectrum. The polarization combiner also has a robust MMI coupler length tolerance of 400 nm. These attributes make it a good candidate for using this proposed device in photonic integrated circuits for improving power ability at the transmitter system.

## 1. Introduction

The integration of photonic devices with electronics has become increasingly important in recent years, leading to a need for compact and low-loss waveguide components [1]. For a high-speed photonic transmitter system, larger RF lines are required to be integrated into the modulator component which leads to more power losses. Thus, significant input power is required to compensate for the overall power losses in the transmitter system [2]. While a laser with a higher power output may be able to meet this power requirement, it may not be a feasible solution for the transmitter system due to potential nonlinear effects that can result in increased noise in the system [3]. A more suitable solution is to use a power combiner waveguide, which can combine two or more waveguides into a single waveguide [4]. The slot waveguide is a unique waveguide that can confine and guide light in TE/TM polarization mode in the slab/slot area, resulting in strong power confinement and light guided by total internal reflection (TIR) [5]. These characteristics make slot waveguides a promising candidate for low power loss waveguide applications such as power combiners or splitters [6]. When combined with a MMI device, slot waveguides can serve as an efficient power combiner to combine waveguide components in a photonic integrated circuit (PIC) [7].

One promising approach to the polarization combiner is based on slot waveguide technology. By introducing a slot or slab into the waveguide core, the effective refractive index can be tuned separately for TE and TM polarizations [8], enabling polarization dependent effects to be controlled. Slot waveguides can be fabricated using standard silicon on insulator technology, making them compatible with electronic/photonic devices and facilitating integration [9]. The excellent properties of the slot waveguide, such as strong light confinement and low power loss, make it a promising candidate for the design of new waveguide devices such as splitters [10], combiners [4], demultiplexers [11,12], and optical amplification [13].

In slot waveguide configuration, the waveguide is structured in the form of a sandwich, where the central layer possesses a lower refractive index compared to the surrounding layers. This waveguide can be referred to as a slotted waveguide, where the slots are stacked on top of one another [5,14]. The TM polarization, which is perpendicular to the layers, is mostly confined in the layer with the lower refractive index (slot layer), while the parallel polarization (TE) is mostly confined in the upper and lower layers, which possess higher refractive indices (slab layers) [15]. Consequently, the effective refractive index for each polarization is fundamentally different, enabling the ability to differentiate according to polarization. The confinement of TM polarization is achieved through the discontinuity of the electric field, and the confinement light level is proportional to the ratio of the dielectric coefficients of the materials [16].

The novelty of this work is to show how it’s possible to utilize the self-imaging effect for obtaining both TE/TM mode on the same MMI coupler and also utilize the slot waveguide technology for achieving a very low optical losses for the TE/TM polarization combiner.

In this paper, we propose a compact polarization MMI combiner using silicon (Si) slot waveguide structures that consist of Si and silica (SiO_2_). MMI operates based on the self-imaging principle where the beam is imaged along the waveguide through the interference of several modes [17,18]. MMI devices are used for many applications such as temperature sensors [19], splitters [20,21], filters [22], and couplers [23]. The polarization MMI Combiner is composed of a central waveguide structure that can support a significant number of modes. The number of waveguides at the inputs and outputs determines the MMI nomenclature as an N × M structure [24]. While self-images can exist in a multi-mode three-dimensional structure, for the purpose of a waveguide analysis, such as ours, it can be assumed that modes are equally distributed throughout the propagation time. This assumption allows for an alternative approach to the problem [24]. The use of slot waveguides in the power combiner based on MMI technology offers several advantages, including low insertion loss, low crosstalk, and high fabrication tolerance [7,24]. The investigation in this new design of a limited modes MMI polarization combiner on a silicon on insulator substrate takes advantage of the polarization-dependent properties of the slot waveguide. The paper shows the performances and tolerances of the device, highlighting its potential for use in integrated photonic circuits and especially for combining TE/TM polarization sources.

## 2. Structure and Theoretical Aspects

According to the self-imaging effect, every wavelength that enters the multimode region of the device produces a direct or mirrored image of itself periodically. The distance from the entry to the point of the first image is called the beat length (L_π_). In our case, the MMI coupler is polarization dependent, which means that the beat length is the function of the TM/TE polarization mode, and it is given by [7]:

(1) $$L_{\pi }=\frac{{4n}_{\mathrm{eff}}W_{e}{}^{2}}{3\lambda }=\left\{\begin{array}{c} \frac{{4n}_{\mathrm{eff}}}{3\lambda }{\left[W_{\mathrm{MMI}}+\left(\frac{\lambda }{\pi }\right)\frac{1}{\sqrt{n_{\mathrm{eff}}{}^{2}-n_{\mathrm{clad}}{}^{2}}}\right]}^{2}\;\;\;\;\;\;\;\;\;\;\;\;\;\;,\;\mathrm{For}\;\mathrm{TE}\;\mathrm{mode}\\ \frac{{4n}_{\mathrm{eff}}}{3\lambda }{\left[W_{\mathrm{MMI}}+\left(\frac{\lambda }{\pi }\right)\left(\frac{n_{\mathrm{clad}}}{n_{\mathrm{eff}}}\right)\frac{1}{\sqrt{n_{\mathrm{eff}}{}^{2}-n_{\mathrm{clad}}{}^{2}}}\right]}^{2}\;,\;\mathrm{For}\;\mathrm{TM}\;\mathrm{mode} \end{array}\right.$$

The effective refractive index n_eff_ and effective width W_e_ of the MMI coupler are important parameters for its performance, with the chosen wavelength λ of 1550 nm. The effective width W_e_ can be calculated using the following equation [7]:

(2) $$W_{e}=\left\{\begin{array}{c} W_{\mathrm{MMI}}+\left(\frac{\lambda }{\pi }\right)\frac{1}{\sqrt{n_{\mathrm{eff}}{}^{2}-n_{\mathrm{clad}}{}^{2}}}\;\;\;\;\;\;\;\;\;\;\;\;,\;\mathrm{For}\;\mathrm{TE}\;\mathrm{mode}\\ W_{\mathrm{MMI}}+\left(\frac{\lambda }{\pi }\right)\left(\frac{n_{\mathrm{clad}}}{n_{\mathrm{eff}}}\right)\frac{1}{\sqrt{n_{\mathrm{eff}}{}^{2}-n_{\mathrm{clad}}{}^{2}}},\;\mathrm{For}\;\mathrm{TM}\;\mathrm{mode} \end{array}\right.$$

**Figure 1:**
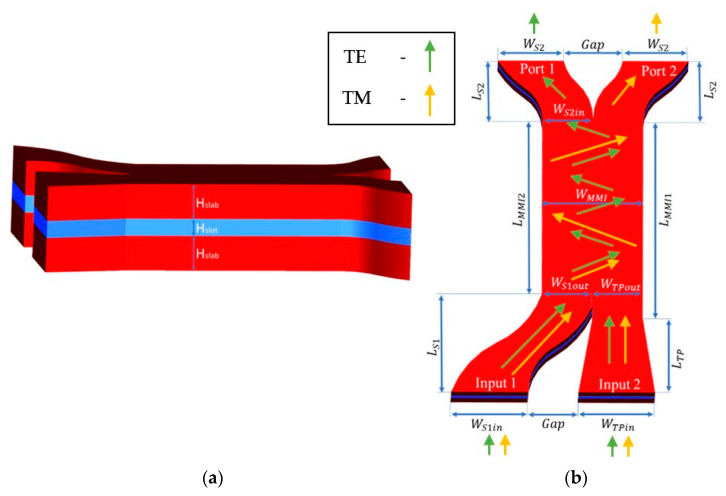
3D sketch of the polarization TE/TM combiner: (**a**) Side view of the slot waveguide structure. (**b**) Top view with arrows that represent the light propagation for TE in green color and TM in orange color.

In order to design the MMI coupler to function as a combiner TE/TM polarization, an asymmetric coupler configuration has been used as shown in Figure 1b with two lengths of L_MMI1_ and L_MMI2_. Thus, the length of the MMI coupler (L_MMI1_) is suitable for both TE/TM polarization beat length and must fulfill the condition given by [25]:

(3) $$L_{\mathrm{MMI}1}\approx pL_{\pi }^{TE}=\left(p+q\right)L_{\pi }^{TM}$$

where *L_π_^TE^* is the beat length for TE polarization, *L_π_^TM^* is the beat length for *TM* polarization, and *p* and *q* are natural numbers that represent the number of transitions of transfer energy inside the MMI coupler.

The strong light confinement in slot waveguide structures is due to the boundary conditions of the electric field at the interface between the low refractive index material SiO_2_ and the high refractive index material Si. The amplitude ratio (AR) of the Electric field can be determined using the following equation [25]:

(4) $$\mathrm{AR}=\frac{E_{\left|x\right|=-\;b}}{E_{\left|x\right|=+b}}=\frac{n_{\mathrm{Si}}^{2}}{n_{{\mathrm{SiO}}_{2}}^{2}}$$

The insertion loss of the device is given by:

(5) $$\mathrm{Losses}\left[\mathrm{dB}\right]=-{10\mathrm{Log}}_{10}\left(\frac{P_{\mathrm{out}}}{P_{\mathrm{in}}}\right)$$

where P_out_ and P_in_ are the output and input powers, respectively.

The Extinction Ratio is given by:

(6) $$\mathrm{Extinction}\;\mathrm{Ratio}[\mathrm{dB}]=\left\{\begin{array}{c} {10\mathrm{Log}}_{10}\left(\frac{P_{\mathrm{TM}}}{P_{\mathrm{TE}}}\right),\;\mathrm{For}\;\mathrm{TE}\;\mathrm{mode}\\ {10\mathrm{Log}}_{10}\left(\frac{P_{\mathrm{TE}}}{P_{\mathrm{TM}}}\right),\;\mathrm{For}\;\mathrm{TM}\;\mathrm{mode} \end{array}\right.$$

where P_TM_ and P_TE_ are the TE and TM mode polarization powers, respectively.

Figure 1a,b illustrate the geometric structure of the TE/TM polarization combiner, in which the light source enters through the two inputs and exits through the outputs Port 1 and Port 2. In Figure 1a,b, red color areas represent Si, and light blue color areas represent SiO_2_. The white color is the cover material that also represents SiO_2_. The component is made of five parts: input taper waveguide, input S-bend, asymmetrical MMI coupler, and two output S-bends. The asymmetrical MMI was used to be suitable for both TE/TM polarization modes with their beat length. Input 1 is an S-bend waveguide, and input 2 is a taper waveguide. Figure 1a demonstrates the slot waveguide layers which are built in a “sandwich” shape, where the outer layers (slab) are made of Si with a refractive index of 3.476, and the inner layer (slot) is made of SiO_2_ with a refractive index of 1.444 for the operated wavelength of 1550 nm.

Figure 1b demonstrates the top view and the dimensions of the polarization combiner. In this figure, green arrows represent the TE light propagation, and orange arrows represent the TM propagation of light. The length of the MMI coupler is 8.15 μm (L_MMI1_) and 5.65 μm (L_MMI2_). Additionally, the width of the MMI coupler is 0.64 μm (W_MMI_). The width of S-bend1 varies from 0.5 μm (W_S1in_) to 0.32 (W_S1out_) with a length of 6.42 μm (L_S1_), and the width of S-bend2 varies from 0.32 μm (W_S2in_) to 0.46 μm (W_S2_) with a length of 0.4 μm (L_S2_). These sizes were selected to minimize the bend loss as much as possible according to Zamhari and Ehsan [26] and still kept a compact device size. The width of the taper varies from 0.5 μm (W_TPin_) to 0.32 (W_TPout_) with a length of 3.92 μm (L_TP_), and these values have been selected to achieve adiabatic taper [27]. Gap is the distance between the inputs and outputs which is 0.43 μm, and this size was selected to avoid light coupling between the output ports. The thickness of the slot is 0.1 μm (H_slot_) and the thickness of the slab is 0.27 μm (H_slab_) as can be shown in Figure 1a.

## 3. Results

**Figure 2:**
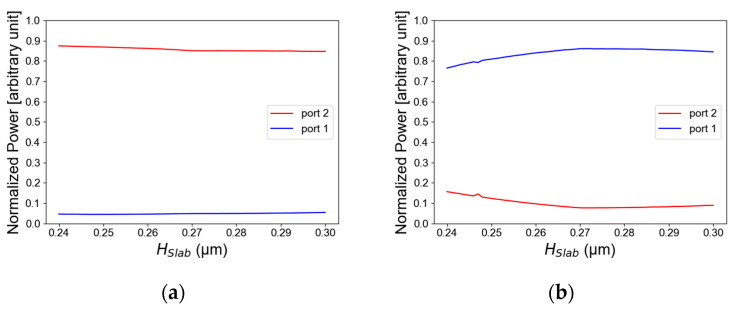
Normalized power as a function of the slab (Si layer) thickness: (**a**) TE mode polarization, (**b**) TM mode polarization.

The simulations of a compact TE/TM polarization MMI combiner using a Si slot waveguide were performed using the Rsoft-cad software, utilizing the full-vectorial beam propagation method (FV-BPM) for numerical solutions of Maxwell’s equations. From the simulations, the optimal parameters for the polarization combiner were set to be H_slot_ = 100 nm, H_slab_ = 270 nm, W_MMI_ = 640 nm, and L_MMI1_ = 8150 nm. Figure 2a,b show the light intensity as a function of the Si layer thickness for TE/TM polarization. In these figures, it can be seen that the optimal value from the simulation is 270 nm for the Si layer thickness. The thickness tolerance range is 240 nm to 300 nm. In TE polarization mode, this range is suitable to transmit more than 80% of the total power as can be seen in Figure 2a, and in TM polarization mode it is suitable to transmit more than 78% of the total power as shown in Figure 2b. These tolerance results are used for avoiding geometrical fabrication errors which can happen. Thus, these results are suitable for using a fab facility with high accuracy of ±30 nm from the optimal value.

**Figure 3:**
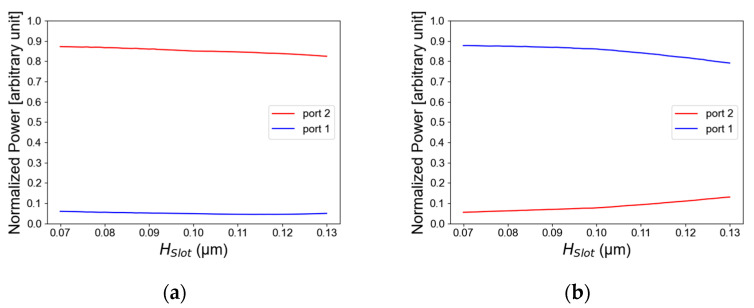
Normalized power as a function of the slot thickness: (**a**) TE mode polarization, (**b**) TM mode polarization.

Figure 3a,b illustrate the light intensity as a function of the SiO_2_ layer thickness for TE/TM polarization. The thickness tolerance range is 70 nm to 130 nm. In TE polarization mode, this range is suitable to transmit more than 80% of the total power as can be seen in Figure 3a, and in TM polarization it is suitable to transmit more than 79% of the total power shown in Figure 3b. The optimal value from the simulation is 60 nm. From the optimizations of Figure 2a,b and Figure 3a,b, the Si slot waveguide dimensions have been found for guiding the light in TM/TE polarization mode. These optimal values have been chosen to support both TE and TM mode solution to be suitable to the configuration of the Si slot waveguide structures and to obtained best performance for the TE/TM polarization combiner.

**Figure 4:**
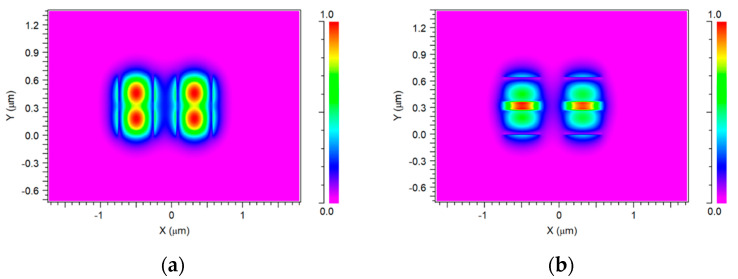
Solution of the TE/TM polarizations for 2 × 1 power combiner: (**a**) TE mode polarization, (**b**) TM mode polarization.

Using the optimal Si slot waveguide parameters, the fundamental mode for TE/TM polarization mode at a 1550 nm-operated wavelength has been solved. Figure 4a shows the TE polarization mode solution for both excited input ports at 1550 nm wavelength for the slot waveguide structure, as can be seen in the red color areas which represent the high light intensity confinement achieved in the Si layers as expected from the physical behavior of the TE mode in the slot waveguide. Figure 4b shows the TM polarization for both laser sources at 1550 nm wavelength for the slot waveguide structure. As shown in Figure 4b, the light is guiding and has strong light confinement through the SiO_2_ layer thickness as can be noticed (red color). From these figures combined with Equation (5), the value has been found to be 5.79.

**Figure 5:**
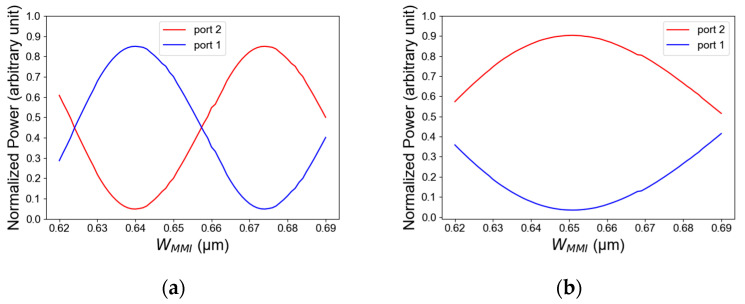
Normalized power as a function of MMI coupler width: (**a**) TE mode polarization, (**b**) TM mode polarization.

Figure 5a,b show the intensity of light as a function of waveguide MMI coupler width for TE/TM polarization mode and the optimization of selecting the optimal MMI width. The width tolerance range for TE polarization is 632 nm to 648 nm, which is suitable to transmit more than 77% of the total power as can be seen in Figure 5a, and the width tolerance of TM polarization is 632 nm to 667 nm which is suitable to transmit more than 80% of the total power shown in Figure 5b. In this study, it can be seen that the error geometrical fabrication for the TE mode is more sensitive than the TM mode. Thus, for achieving light guiding in both modes TE and TM polarization, an optimal MMI width value was selected to be 640 nm.

**Figure 6:**
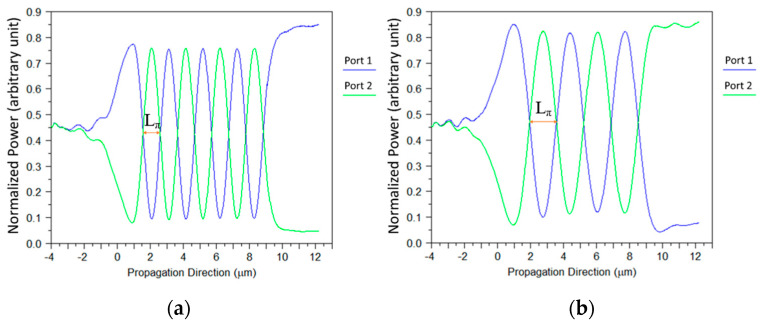
Light coupling between two close segments: (**a**) TE mode polarization, (**b**) TM mode polarization.

A simulation of the transfer energy between the optical path of port 1 and port 2 inside the MMI coupler has been conducted to extract the beat length size. The light coupling mechanism of the self-imaging effect of the two combined sources inside the MMI coupler under the TE polarization mode at 1550 nm wavelength as shown in Figure 6a and for the TM polarization mode can be seen in Figure 6b. According to the calculations from these figures, the beat length at TE polarization mode is 1140 nm as shown in Figure 6a, and for TM polarization mode the beat length is 1720 nm as shown in Figure 6b.

**Figure 7:**
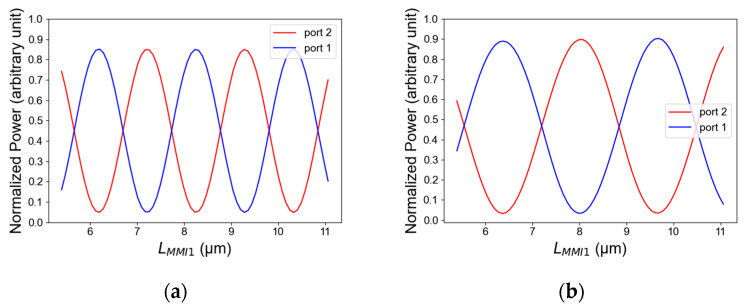
Normalized power as a function of MMI coupler length: (**a**) TE mode polarization, (**b**) TM mode polarization.

Figure 7a,b show the light intensity as a function of the MMI coupler length (L_MMI1_) for TE/TM mode polarization. From these figures, the suitable MMI coupler length that fulfills the condition of Equation (3) for both polarization modes TE and TM can be found, and its optimal value is 8150 nm (L_MMI1_); thus, the value of L_MMI2_ can be easily found, and its value is 5650 nm. In these figures, it can be noticed that the length tolerance range is 7740 nm to 8630 nm. In both polarization modes, this range is suitable to transmit more than 75% of the total power in TE mode as shown in Figure 7a, and in TM mode polarization it is suitable to transmit more than 80% of the total power as shown in Figure 7b. From the tolerance results, it can be studied that the MMI coupler length has a good stability for geometrical fabrication errors with a high accuracy of at least ±400 nm from the optimal value. It can be seen by the results showing in Figure 7a,b that TE polarization mode has a short beat length compared to TM mode and therefore is more sensitive to changes in the MMI coupler length.

**Figure 8:**
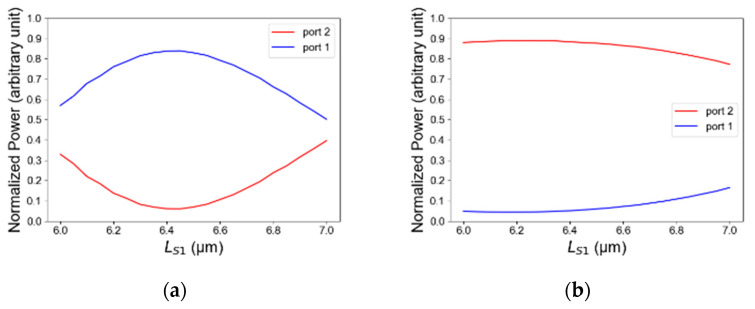
Normalized power as a function of input 1 length: (**a**) TE mode polarization, (**b**) TM mode polarization.

Figure 8a,b show the light intensity as a function of the length of S-Bend1 for both TE/TM polarizations. The length tolerance range is 6250 nm to 6590 nm. In TE polarization, this range is suitable to transmit more than 80% of the total power as shown in Figure 8a, and in TM polarization it is suitable to transmit more than 88% of the total power as can be seen in Figure 8b. The optimal value from the simulations is 6420 nm which is suitable for both mode operations of TE and TM. The tolerances results are good for a fab facility with high accuracy of at least ±200 nm from the optimal value. Thus, the length of S-bend1 has a robust tolerance for the fabrication process, and the same physical behavior is obtained for S-bend2.

**Figure 9:**
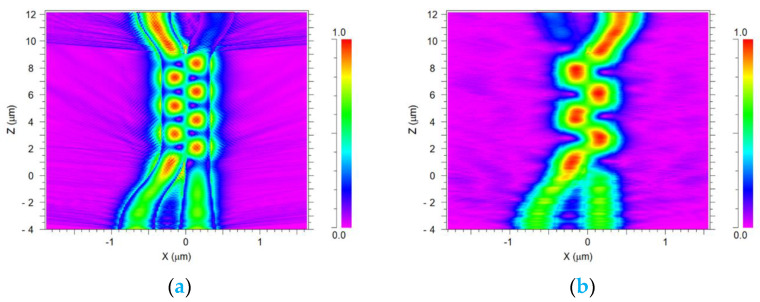
Light intensity propagation for the polarization combiner at 1550 nm wavelength: (**a**) TE mode polarization, (**b**) TM mode polarization.

Figure 9a,b show the light intensity propagation progress of the combiner polarization for each TE/TM mode at 1550 nm wavelength. These figures illustrate the control of light coupling between two closely located slot waveguide units and show how the light of two sources can be combined. It can be seen in Figure 9a that there are seven light energy transfers in TE mode, and the combiner efficiency is 83.8% and in Figure 9b can be seen four light energy transfers in TM mode, and the combiner efficiency is 88.5%. It is important to emphasize that these transfer energy results are suitable for fulfilling the condition in Equation (3). Using these results combined with Equation (5), the light coupling losses can be found and are 0.76 dB and 0.56 dB for TE and TM polarization modes, respectively. Additionally, the extinction ratio has been calculated using Equation (6), and the results are 10.94 dB and 13.08 dB for TE and TM modes, respectively.

**Figure 10:**
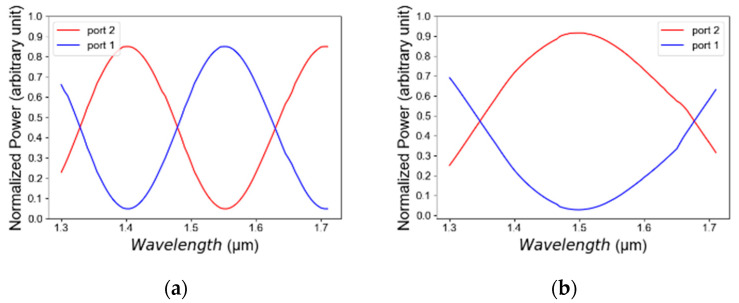
Normalized power as a function of wavelength: (**a**) TE mode polarization, (**b**) TM mode polarization.

Figure 10a,b illustrate the light intensity as a function of the operated wavelength for TE/TM mode polarizations. The wavelength tolerance range is 1480 nm to 1605 nm, in TE mode polarization, and this range is suitable to transmit more than 60% of the total power as shown in Figure 10a. In TM mode polarization, it is suitable to transmit more than 72% of the total power as shown in Figure 10b. Thus, the proposed TE/TM combiner can function well over the C-band spectrum (1530–1570 nm) with an overall 90% of the total power as shown in Figure 10a,b.

**Figure 11:**
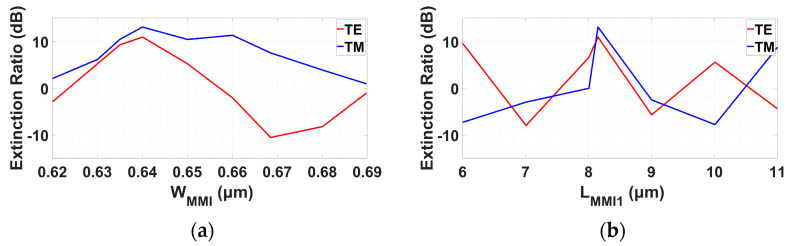
Extinction ratio as a function of width and length polarization TE/TM combiner: (**a**) W_MMI_, (**b**) L_MMI1_.

Figure 11a,b show the extinction ratio as a function of W_MMI_ and L_MMI1_, respectively, for TE and TM modes. According to the tolerance in Figure 5a,b, the extinction ratio in Figure 11a changes accornigly in range of −3 dB to −2.5 dB while the optimal point is at 10.94 dB for TE mode. For TM mode, the range is 2 dB to 11.02 dB, and the optimal point is at 13.08 dB. The extinction ratio in Figure 11b changes in accordance to the tolerance Figure 6a,b. For TE mode, the range is 3 dB to 4 dB while the optimal point is at 10.94 dB. For TM mode, the range is −1.95 dB to 6 dB, and the optimal point is at 13.08 dB.

In Table 1, a comparison is shown between the proposed TE/TM polarization combiner design introduced above and other TE/TM polarization splitter/combiner previously proposed in published papers. As shown in Table 1, the main characteristics of the TE/TM polarization combiner that were compared are waveguide technology, footprint size (length (L) × width (W) × height (H), losses (dB), extinction ratio (dB), and PIC capable.

From Table 1, it can be noticed that the main benefits of the proposed TE/TM polarization combiner are the compact size and the low losses compared to other waveguide techniques. This compact size is especially advantageous in applications where size constraints or space limitations are critical factors. Furthermore, waveguides exhibit low losses, meaning that there is minimal attenuation or energy loss during wave transmission. This characteristic ensures high signal integrity and reliability, making the waveguide in this work highly desirable in applications that demand low loss and high-quality signal transfer.

From particle view, the main loss model of the light fabricated by lithography is the scattering light loss induced by waveguide sidewall roughness. To reduce this effect, it is better to use a horizontal slot waveguide and to apply some process [31,32,33]. Additionally, in our case because of the compact configuration, the light scattering is negligible.

The proposed device can be fabricated using the standard fabrication of slot waveguide which can be described in four steps. For the first step, the waveguide patterns are generated on a wafer via a conventional lithography technique. At the second step, the patterns are transferred into the top later with an inductively coupled plasma reactive ion etching process. At the third step, the photoresist used for pattern generation is removed by acetone and plasma cleaning. At the fourth step, the undercut of oxide cladding is performed by isotropic buffered oxide etch [34].

## 4. Conclusions

In this research, we have demonstrated that it is possible to combine two coherent laser sources under the TE and TM polarization over the C-band spectrum using a single MMI coupler based on Si slot waveguide technology for achieving a higher intensity level. This study shows how to design a double Si slot waveguide structure for obtaining the fundamental mode solution for both TE and TM modes with strong light confinement at the slot area (TM mode) and slab areas (TE mode). The advance of this technology shows that a compact beat length of 1.14 µm for TE mode and 1.72 µm for TM mode can be obtained in the MMI coupler which leads to a short light coupling length for transferring the energy of the combined signals by utilization of the self-imaging effect. The device was solved through the FV-BPM algorithm, and results show that after a short coupling length of 16.15 µm the polarization combiner efficiency reaches up to 83.8% for TE mode and 88.5% for TM mode at 1550 nm wavelength. In addition, a good extinction ratio of 10.94 dB for TE polarization and 13.08 dB for TM polarization was obtained with low insertion losses of 0.76 dB in TE polarization and 0.56 dB in TM polarization. The tolerance analysis shows a good flexibility range for the MMI coupler size and the Si slot waveguide layer structures and especially for the MMI coupler length with a ±400 nm shifting over the optimal value. These tolerances results can be used in a good fab facility with high accuracy for easy fabrication of the proposed device. The design presented has the advantage of a lack of need for calibration and is implemented optimally in PIC. Compared to other options, the proposed combiner with lower losses and a significantly smaller structure than other technologies, which allows the use of PIC while minimizing them, thereby increasing the power level without compromising the quality of the signal. Additionally, this study can be used for the combination of two coherent TE/TM polarization C-band laser sources that are integrated into a high-speed transmitter system. This research can be utilized to better realize how to combine various coherent laser sources at the C-band spectrum under the TE/TM polarization to improve transmitter system power performances. A heater or PIN phase shifter can be added to the waveguide taper input to adjust the phase to be suitable for the modulation system.

## Figures and Tables

**Table 1:** Comparison between the main characteristics of different TE/TM mode polarization splitter/combiner.

Waveguide Technology	Size (L × W × H) µm^3^	Losses (dB)	Extinction Ratio (dB)	PIC Capable
MMI-based polarization beam splitter with A sub-wavelength grating (SWG) structure [28]	21 × 4.8 × 0.4	TE: 1.25 TM: 0.9	TE: 18 TM: 17.3	Yes
2 × 2 PBS based on SWG MMI coupler for the silicon-on-insulator platform [29]	92.5 × 2.5 × 0.5	TE: 1.9 TM: 2.5	TE: 14.5 TM: 11.7	Yes
MMI-based PBS on a Si strip waveguide [30]	132.64 × 4.2 × 0.22	TE: 1.2 TM: 2.2	TE: 22.7 TM: 30.6	Yes
Slot waveguide (in this work)	16.15 × 1.6 × 0.64	TE: 0.56 TM: 0.76	TE: 10.94 TM: 13.08	Yes
